# 3D-based strain analysis and cardiotoxicity detection in cancer patients received chemotherapy

**DOI:** 10.1186/s12885-023-11261-y

**Published:** 2023-08-16

**Authors:** Muhammad Azzam, Mohammad Wasef, Hani Khalaf, Ahmed Al-Habbaa

**Affiliations:** 1 Cardiology department, Faculty of Medicine, Al-Azhar University, Cairo, Egypt; 2 Queen Alexandra Hospital, Portsmouth, UK

**Keywords:** Chemotherapy, Cardiotoxicity, Cancer, 3D, Strain

## Abstract

**Background:**

Chemotherapy-induced cardiotoxicity has become a prevalent complication. Regular monitoring of patients who received chemotherapy using 3D strain parameters may aid in early detection of myocardial damage and its prevention. The purpose of this study was to evaluate the effectiveness of three-dimensional speckle tracking imaging (3D-STI) in diagnosing and predicting the likelihood of cardiotoxicity. This was achieved by conducting a systematic review of original research articles.

**Objectives:**

To evaluate the role of 3D speckle tracking echocardiography in early detection of cardiotoxicity.

**Methods:**

Relevant case control studies published prior to December 2022 were extracted to assess cardiotoxicity by 3D STE in patients after chemotherapy.

**Results:**

A total of 1991 chemotherapy treated patients and control patients were included in the present review via pooling 22 studies.

**Conclusions:**

3D speckle tracking echocardiography has the utility of non-invasive and objective evaluation of changes in left ventricular function in cancer patients undergoing chemotherapy.

**ROSPERO registration No:**

Study ID, CRD42023383790 on PROSPERO: International prospective register of systematic reviews.

## Background

Millions of people are fighting against cancer as one of the leading causes of death [1]. Over the last decade, the number of cancer survivors has dramatically increased, mainly due to advances in chemotherapy and imaging modalities [2]. Adverse effects of chemotherapy, especially cardiac ones can lead to high morbidity and even death among cancer survivors [3].

Cardiotoxicity often leads to treatment discontinuation with a subsequent increased risk of cancer recurrence and mortality, which has been reported to be as high as 40% over the next 5 years following chemotherapy [4]. The extent of cardiotoxicity stands on the regimen of treatment utilized, cancer type and underlying patient risk factors [5].

Left ventricular ejection fraction (LVEF) values are used in practice to assess and follow patients with susceptible myocardial toxicity but the accuracy and sensitivity in determining myocardial injury at an early stage have lower sensitivity and high variability. Once LVEF declines are detected, advanced myocardial injury has occurred and is likely irreversible [6, 7].

There are advancements in screening modalities to diagnose subclinical myocardial dysfunction. Speckle tracking allows comprehensive quantitative evaluation of LV deformation in terms of strain and strain rate [8]. Post-therapeutic left ventricular two-dimensional longitudinal strain (GLS) reduction greater than 15% in patients with LVEF greater than 50% has been regarded as subclinical myocardial injury [9–12].

Even this threshold still may not be enough to allow early detection of cancer therapy related cardiac dysfunction (CTRCD). Further inclusion of other strain parameters may facilitate comprehensive evaluation of myocardial mechanics that can help in detecting subclinical cardiotoxicity [13].

Three-dimensional speckle tracking imaging (3D-STI) represents an advanced form of echocardiographic technology for detection of myocardial injuries at earlier stages. 3D-STI has higher measurement reproducibility with tracking of out-of-plane speckle motion [14]. Since multi section images during the same cardiac cycle can be resolved, the evaluation time is reduced with higher sensitivity and detectability at earlier stages of cardiotoxicity [15].

Left ventricle has garnered much attention from researchers, however, right ventricle (RV) evaluation by STE has been recognized as an independent prognostic factor and an asset in recognizing early subclinical changes in the myocardium for routine follow-up in cancer patient populations [16, 17].

Monitoring of chemotherapy related cardiac dysfunction is not only for patients currently under therapy but also for cancer survivors who remain at risk of developing adverse events even after treatment completion, which can last for years [18]. Therefore, it is of paramount importance to precisely identify cancer survivors at high risk of developing cardiotoxicity and to assess our tools for early detection.

This systematic review aims to assess and monitor subclinical cardiotoxicity detection using different 3D strain parameters in cancer patients during and after chemotherapy.

## Methods

### Search strategy

**Fig. 1:**
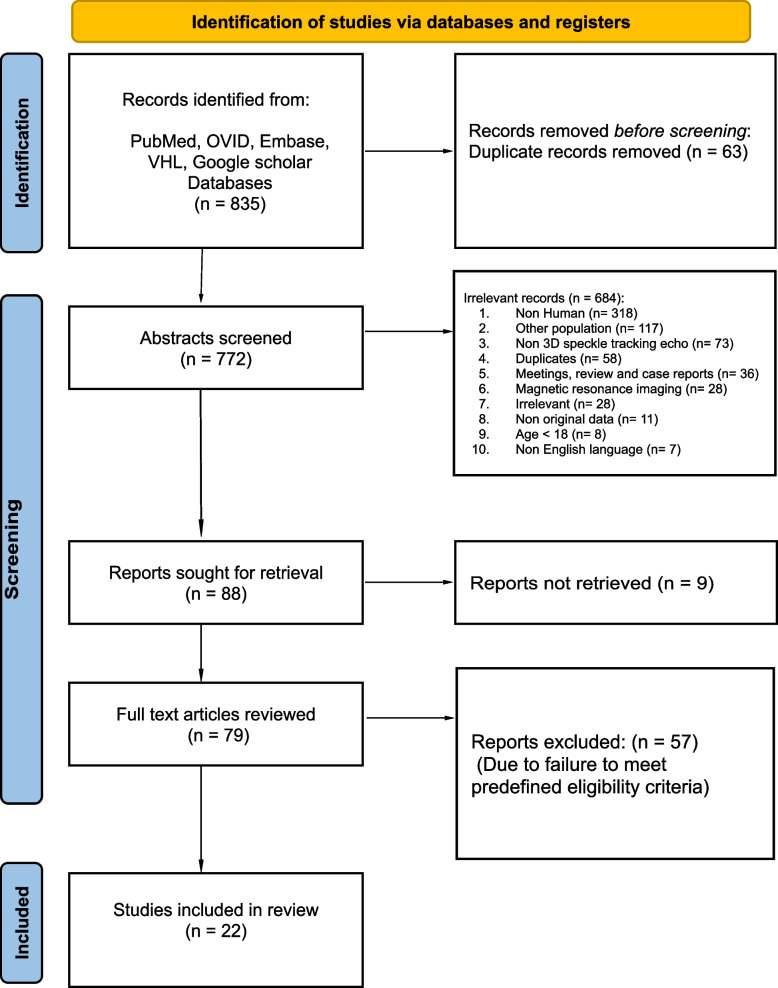
PRISMA 2020 flow diagram Page MJ, McKenzie JE, Bossuyt PM, Boutron I, Hoffmann TC, Mulrow CD, et al. The PRISMA 2020 statement: an updated guideline for reporting systematic reviews. BMJ 2021;372:n71. https://doi.org/10.1136/bmj.n71

This is a systematic review performed according to the Preferred Reporting Items for Systematic Reviews (PRISMA) guidelines [19]. A literature search for potential studies published until December 2022 was done using PubMed, OVID, VHL, Web of science, Cochrane, Embase and Google Scholar. The details of the search are summarized in Fig. 1. University review board approval was not required for this study.

### PICO criteria

#### Population

Cancer patients aged 18 years or older who had undergone chemotherapy and had recorded 3D strain parameters, with no restrictions on gender or ethnicity. Participants must also have no evidence of cardiac symptoms or signs, no history of cardiomyopathies, uncontrolled cardiac risk factors, or significant valvular heart disease.

#### Intervention

Any chemotherapy is known to cause cardiotoxicity.

#### Comparison

3D based strain parameters in patients who developed and those who did not develop cancer therapy related cardiovascular toxicity.

#### Outcome

Value of 3D based speckle tracking echocardiography parameters in detecting cardiac affection.

### Inclusion and exclusion criteria

Inclusion criteria were as follows: Adult (≥ 18 years) cancer patients undergoing chemotherapy were included. We included all clinical studies (randomized controlled trials (RCTs), prospective or retrospective observational studies) investigating the ability of 3D speckle tracking based strain analysis to detect cardiotoxicity and cardiac adverse events regardless of its definition. Non-English case reports and articles were not included.

### Study selection

Abstracts and full texts were reviewed in a blind standardized manner. Relevant studies from references in selected papers were scanned for possible inclusion. RAYYAN software was used. Before final approval, any disagreements between researchers were resolved by senior contributors.

### Data extraction

Two authors (MA and MA) extracted the data, using predefined standardized data extraction form. Data extraction included: study characteristics as design and duration, study population as total number of included patients and type of cancer being treated, cardiac therapy related cardiac dysfunction (CTRCD) definition, CTRCD events, investigated 3D speckle tracking based parameters, methods of investigations and results. Microsoft Office Excel was used for data extraction.

### Bias risk assessment, quality, and validity of included studies

The included studies were assessed by four reviewers for risk of bias and quality, including the use of the Newcastle–Ottawa Scale [20] (Table 1).

**Table 1:** Newcastle–Ottawa Scale

**PAPERS**	**Country**	**Year**	**JOURNAL**	**NEWCASTLE–OTTAWA SCALE** ** (TOTAL SCORE)**
**ALAM ET AL.**	India	2019	INDIAN HEART JOURNAL	**8**
**CHEN ET AL.**	China	2019	ECHOCARDIOGRAPHY	**8**
**CHEN ET AL.**	China	2022	JOURNAL OF HEALTHCARE ENGINEERING	**8**
**CRUZ ET AL**	Portugal	2019	CLINICAL RESEARCH IN CARDIOLOGY	**8**
**GUAN ET AL.**	Italy	2021	FRONTIERS IN PHARMACOLOGY	**8**
**HONG.K ET AL**	China	2013	JOURNAL OF THE AMERICAN SOCIETY OF ECHOCARDIOGRAPHY	**7**
**HOU ET AL.**	China	2018	JOURNAL OF HAINAN MEDICAL UNIVERSITY	**7**
**KARAKULAK ET AL**	Turkey	2020	CARDIOVASCULAR TOXICOLOGY	**6**
**LI. ET AL.**	China	2019	INTERNATIONAL JOURNAL OF CARDIOVASCULAR IMAGING	**6**
**MIHALCEA ET AL.**	Romania	2020	NATURE, SCIENTIFIC REPORTS	**8**
**MIYOSHI. T ET AL.**	Jaban	2014	ECHOCARDIOGRAPHY	**7**
**MORNOS C ET AL.**	Greece	2014	HELLENIC JOURNAL OF CARDIOLOGY	**8**
**PIVETA. R ET AL.**	Brazil	2022	FRONTIERS IN CARDIOVASCULAR MEDICINE	**8**
**SANTORO ET AL.**	Italy	2017	EUROPEAN HEART JOURNAL CARDIOVASCULAR IMAGING	**8**
**SONG F ET AL.**	China	2017	CARDIOLOGY JOURNAL	**7**
**SONG F ET AL.**	China	2017	INTERNATIONAL JOURNAL OF CARDIOVASCULAR IMAGING	**7**
**WANG, Y. ET AL.**	China	2020	INTERNATIONAL JOURNAL OF CARDIOVASCULAR IMAGING	**8**
**WANG, Y. ET AL.**	China	2020	INTERNATIONAL JOURNAL OF CARDIOVASCULAR IMAGING	**8**
**XU ET AL.**	China	2019	INTERNATIONAL JOURNAL OF CARDIOVASCULAR IMAGING	**8**
**XU ET AL.**	China	2021	INTERNATIONAL JOURNAL OF GENERAL MEDICINE	**8**
**ZHANG ET AL.**	USA-	2018	JACC: CARDIOVASCULAR IMAGING	**9**
**ZHAO ET AL.**	China	2020	JACC: CARDIOONCOLOGY	**8**

## Results

The literature research retrieved 835 studies. After the removal of duplicates, 772 studies remained. After the review of all abstracts, another 684 studies were excluded due to irrelevance. The remaining 79 articles underwent full reading and 19 met the predefined inclusion criteria. Three more studies were included from the bibliography scanning making a total of 22 papers included in the present review (Fig. 1). Overall, this systematic review included 1991 patients, among 22 non-RCT studies. Details of the included studies are reported in (Tables 2 and 3).

**Table 2:** Summary of included papers

**REFERRENCES**	**COUNTRY**	**YEAR**	**NUMBER**	**AGE** **(YEARS)**	**TYPE OF CANCER**	**CHEMOTHERAPY PROTOCOL**	**VENDOR**	**WORK STATION**
**ALAM ET AL.**	India	2019	46 patients	44.17 ± 10.95	Various tumour types	Doxorubicin regimen	Vivid E9, GE	EchoPac PC
**CHEN ET AL.**	China	2019	83 patients	49.25 ± 8.75	Breast cancer	Epirubicin + cyclophosphamide regimen	GE Vivid E9	EchoPac PC
**CHEN ET AL.**	China	2022	39 patients	52.63 ± 9.28	Multiple myeloma	6 PAD regimen (bortezomib + Doxorubicin + dexamethasone)	Vivid E9, GE	EchoPac PC
**CRUZ ET AL**	Portugal	2019	105 patients	53.8 ± 12.5	Breast cancer	Anthracycline regimen	GE Vivid E9 orE95	EchoPac PC
**GUAN ET AL.**	Italy	2021	79 patients	48 ± 11.5	Breast cancer	Different chemotherapeutic regimens	GE Vivid E95	EchoPac PC
**HONG.K ET AL**	China	2013	53 survivors and 38 control	18.6 ± 5.1	Various tumour types	Anthracycline regimen	Artida, Toshiba	Advanced cardiology Package, Toshiba Medical Systems
**HOU ET AL.**	China	2018	58 patient- and 50 control	45–65	Lung cancer	Pemetrexed + cisplatin regimen	Philips X3-1	TomTec 4D LV analysis
**KARAKULAK ET AL**	Turkey	2020	37 patients and 50 control	53.5 ± 11.5	Chronic Myeloid Leukaemia	Dasatinib or nilotinib after imatinib failure	GE Vivid E9	Echo Pac PC
**LI. ET AL.**	China	2019	83 survivors and 42 control	25.6 ± 6.1	Various tumour types	Anthracycline regimen	Vivid, E95	Echo Pac PC
**MIHALCEA ET AL.**	Romania	2020	110 NHL patients	58 ± 11	Non-Hodgkin’s lymphoma	CHOP (cyclophosphamide, doxorubicin, vincristine, prednisone) regimen	GE Vivid E9	Echo Pac PC
**MIYOSHI. T ET AL.**	Jaban	2014	50 survivors and 20 control	54 ± 15	Various tumour types	Anthracycline regimen	Artida, Toshiba	Advanced cardiology Package, Toshiba Medical Systems
**MORNOS C ET AL.**	Greece	2014	59 patients	50 ± 12	Various tumour types	Anthracycline regimen	GE Vivid E9	Echopac PC
**PIVETA. R ET AL.**	Brazil	2022	51 patients	50.6 ± 11	Breast cancer	(Doxorubicin + cyclophosphamide + trastuzumabin) regimen	Toshiba Artida	Advanced cardiology Package, Toshiba Medical Systems
**SANTORO ET AL.**	Italy	2017	100 patients	48.6 ± 11.1	Breast cancer	ANT and cyclophosphamide and/or 5-fluorouracil for 3–4 cycles	GE Vivid E9	Echopac PC
**SONG F ET AL.**	China	2017	101 patients	49	Large B cell lymphoma	R- CHOP (cyclophosphamide, doxorubicin, vincristine, prednisone) regimen	IE 33, philips	TomTec 4D LV analysis
**SONG F ET AL.**	China	2017	89 Patients	20 – 78	Diffuse non-Hodgkin lymphoma	Anthracycline regimen	IE 33, philips	TomTec 4D LV analysis
**WANG ET AL.**	China	2020	64 patient 32 of them as a control group	33.2 ± 8.3	Breast cancer	Regular CTF followed by dexrazoxane and control group without dexrazoxane	Toshiba Artida SSH-880CV	Advanced cardiology Package, Toshiba Medical Systems
**WANG, Y. ET AL.**	China	2020	30 patients	37—64	Colorectal cancer	mFOLFOX6 regimen	GE Vivid E9	Echopac PC
**XU ET AL.**	China	2019	60 patients	49.3 ± 12.5	large B-cell lymphoma	R- CHOP regimen	IE 33, Philips	TomTec 4D analysis
**XU ET AL.**	China	2021	95 patients	53.2 ± 8.7	Breast cancer	Epirubicin	GE Vivid E9	Echopac PC
**ZHANG ET AL.**	USA-	2018	142 patient and 21 control	41—56	Breast cancer	Doxorubicin + cyclophosphamide followed by paclitaxel with or without trastuzumab	GE Vivid E9 or E7	TomTec 4D LV analysis
**ZHAO ET AL.**	China	2020	74 patients	48.9 ± 11.8	Diffuse large B cell lymphoma	Anthracycline regimen	IE 33, Philips	TomTec 4D RV analysis

*AS* (Arterial stiffness), *CHOP* (Cyclophosphamide, doxorubicin, vincristine, prednisone), *EF* (Ejection fraction), *FW LS* (Free Wall Longitudinal Strain), *GAS* (Global area strain), *GCS* (Global circumferential strain), *GLS* (Global Longitudinal Strain), *GRS* (Global radial strain), *RV* (right ventricle), *RVLFS* (Right ventricle longitudinal free wall strain) and *RVLSS* (Right ventricle longitudinal septal strain)

**Table 3:** Summary of included papers

**REFERRENCES**	**3D STI PARAMETERS**	**ECHO TIMING**	**KEY FINDING**	**PROGNOSIS**
**ALAM ET AL.**	GLS, GCS, GRS, GAS, and 3D EF	baseline and after 4 cycles	all 3D strain parameters were significantly reduced after chemotherapy	No patients developed CTRCD
**CHEN ET AL.**	GAS, GLS, GCS and GRS	4 groups at 0,120 mg/m, 240 mg/m and at the end of chemotherapy 360 mg/m2	GAS derived from 3D‐STI was more accurate and sensitive in responding to myocardial damage than other strain parameters and LVEF with significant negative correlation with anthracycline doses	No patients developld CTRCD
**CHEN ET AL.**	**LV** GLS**, GCS, GRS and RV GLS, GCS, GRS**	Baseline and after cycle 2, 4 and 6	RVGCS, RVGLS, RVGRS, LVGLS, and LVGRS in significantly reduced in patients before and after chemotherapy	No patients developed CTRCD
**CRUZ ET AL.**	GLS, GCS, GRS, and GAS	baseline, during and after chemotherapy	significant worsening of all 3D strain parameters during chemotherapy. Variations of 3D GCS and 3D GRS had a good discrimination for predicting CTRCD	24 patients developed CTRCD during follow up
**GUAN ET AL.**	GLS, GCS, GAS, and GRS	before chemotherapy and after the cycle 2 (T2), cycle 4 (T4), cycle 6 (T6), and cycle 8 (T8)	all 3D strain parameters were significantly reduced at every stage of chemotherapy	9of 79 patent developed CTRCD at different stages of chemotherapy
**HONG.K ET AL.**	Global and segmental strain, SDI, GPI, and Torsion	survivors off treatment for more than 1 year	all 3D strain parameters were significantly reduced in cancer survivors	No patients developed CTRCD
**HOU ET AL.**	GLS, GCS, GRS, GAS,	at 3 cycles of chemotherapy,	3D GAS after three cycles of the chemotherapy group was significantly lower than that of the control group and changes in 3D GAS correlated with oxidative stress and apoptosis markers	No patients developed CTRCD
**KARAKULAK ET AL.**	GLS, GCS, GAS and GRS	after imatinib failure and patient on dasatinib or nilotinib	significant reduction in 3D strain parameters in area with no difference between dasatinib and nilotinib groups. Additionally, area and radial strain had a stronger association with the duration of dasatinib treatment	No patients developed CTRCD
**LI. ET AL.**	GLS, GCS, GRS, GAS, EF	survivors off treatment for 16.0 ± 6.1 years	all 3D strain parameters were significantly reduced in cancer survivors and GLS is considered the most sensitive parameter in the detection of subclinical cardiotoxicity	No patients developed CTRCD
**MIHALCEA ET AL.**	GLS, GCS, GAS and GRS	at baseline, after third cycle and chemotherapy completion	3D strain echocardiography showed a significant decrease of all deformation parameters LS, CS, RS, and AS in the study group after 3rd cycle and persistent after final cycle of therapy, with more important reduction in patient who developed cardiotoxicity. LS were identified as the best independent predictors for 3D LVEF decrease at the end of chemotherapy	18 patients (16%) (group I) developed cardiotoxicity
**MIYOSHI. T ET AL.**	GLS, GCS, GRS and GAS	anthracycline treated patients 13 ± 22 months after treatment	Only 3D-GAS and peak 3D global circumferential strains of the anthracycline group were significantly worse than those of the control group and 3D GAS was the only parameter that could be independently linked to the cumulative doxorubicin dose	No patients developed CTRCD
**MORNOS C ET AL.**	GLS, GCS and GRS	before, and at 12 weeks after anthracycline treatment	3D GLS, 3D GCS, and 3D GRS revealed significant changes 12 weeks after chemotherapy and 3D GLS emerged as the only independent predictor of later cardiotoxicity	Eight patients (13.5%) developed cardiotoxicity
**PIVETA. R ET AL.**	GLS, GRS, GAS, GCS, rotation, torsion, and twist	baseline, after 120 mg &240 mg of doxorubicin, after 6 and 12 months	After a lower cumulative dose of doxorubicin (120 mg/m2), 3D GAS was the only parameter that was changed and was associated with a subsequent decrease in LVEF. while most myocardial deformation parameters: 3D GLS, 3D GRS and 3D GCS significantly changed after the cumulative dose of 240 mg/m2 of doxorubicin and no changes in rotation, torsion, or twist in any of the evaluation stages,	(13%) 7 patients presented developed significant decrease in LVEF during follow up
**SANTORO ET AL.**	GLS, GCS, GAS and GRS	before and after completion of ANT chemotherapy	all 3D strain parameters were significantly reduced after chemotherapy with the greatest effect for GCS and GAS	No patients developed CTRCD
**SONG F ET AL.**	Apical and basal rotation, twist and torsion	baseline, 2, and 4 cycles	3D LV apical rotation, basal rotation, twist, and torsion declined progressively during the whole procedure (baseline vs. two and four cycles of the regimen	No patients developed CTRCD
**SONG F ET AL.**	GLS, and GCS and **RV GLS**	baseline, 4 cycles and at the end	3D GLS and GCS of LV and GLS of RV decreased significantly after four cycles of the therapy	No patients developed CTRCD
**WANG ET AL.**	**RV GLS, RV GCS, RV GRS and RV GAS**	before and after completion of chemotherapy	RV GLS and RV GAS were significantly reduced after chemotherapy	No patients developed CTRCD
**WANG, Y. ET AL.**	GLS, GCS, GRS, GAS, MCI and LVtw	Baseline, and after 1, 6 and 12 cycles	3D STE parameters GLS, GAS, MCI and LVtw decreased after the first cycle of chemotherapy. Increasing cumulative dose of mFOLFOX6 correlated with decreases in left ventricular MCI, GLS, GAS, GCS, GRS, and LVtw, with statistically significant differences between pre- and post-chemotherapy. In particular, the decrease in MCI was found to be the most significant	No patients developed CTRCD
**XU ET AL.**	3D-GLS, 3D-GCS, 3D-LS of all segments	baseline, after the completion of two cycles and four cycles of the regimen respectively	3D-GLS reduced significantly after four cycles of anthracycline, 3d-GCS showed no significant changes during the whole study. All longitudinal strains in the middle and apical segments showed a significant decrease after four cycles with an early drop of apical anterior and septal wall LS after two cycles	No patients developed CTRCD
**XU ET AL.**	GLS, **RV GLS, and RV FW LS**	at baseline, the end and 12 months after chemotherapy	3D GLS of LV and GLS and FWLS of RV decreased significantly at 12 months after chemotherapy	(10.5%) 10 patients developed Subclinical CTRCD during follow-up. Compared to baseline
**ZHANG ET AL.**	GCS, GLS, principal strain, twist, and torsion	before, during and annually after termination in patents received anthracycline and participants treated with trastuzumab underwent echocardiograms every 3 months during therapy and annually thereafter	After a median 2.1 years follow-up period they reported that 3D LVEF, GCS, and GLS, had initial decrement. In contrast, values for 3D twist and torsion plateaued at six months without substantial subsequent change 3DLVEF, GCS, GLS, and principal strain were associated with concurrent and subsequent changes in systolic function	No patients developed CTRCD
**ZHAO ET AL.**	**RVLFS and RVLSS** **LV GLS, LV GCS**	baseline, 2, 4, and 6 cycles	3D RV LFS and longitudinal septal strain LV GLS were statistically significant before and after 4 cycles of chemotherapy and only RV LFS was associated with subsequent RV cardiotoxicity with RVEF decline at the end of follow-up. However, RV LSS and LV GCS were only significantly decreased at T3	27 patients developed cardiotoxicity after 6 cycles of chemotherapy (T3)

*AS* (Arterial stiffness), *CHOP* (Cyclophosphamide, doxorubicin, vincristine, prednisone), *EF* (Ejection fraction), *FW LS* (Free Wall Longitudinal Strain), *GAS* (Global area strain), *GCS* (Global circumferential strain), *GLS* (Global Longitudinal Strain), *GRS* (Global radial strain), *RV* (right ventricle), *RVLFS* (Right ventricle longitudinal free wall strain) and *RVLSS* (Right ventricle longitudinal septal strain)

The following points are going to be highlighted in the upcoming sections:

1. 3D speckle tracking echocardiography in breast cancer patients.
2. 3D speckle tracking echocardiography in non-breast cancer patients:
  A Pediatric cancer survivors.
  B Non breast cancer tumors with different chemotherapy regimens.

### 3D speckle tracking echocardiography in breast cancer patients

Breast cancer is the first cancer worldwide in terms of cases number [21]. Regarding detection of cardiotoxicity, it is the most extensively studied cancer [22]. Echocardiography is the most used investigation for the follow-up of patients during cancer therapy with known cardiotoxic effect. 2D echocardiography is a widely available tool for estimating the left ventricular ejection fraction, which is the parameter used so far for detection of cardiotoxicity. With the development of 3D echocardiography, estimation of LVEF by 3D volume calculation is gaining popularity being more accurate and reproducible [23]. Speckle tracking echocardiography appeared as an attractive technique for calculation of left ventricular global longitudinal strain which can detect early myocardial affection before affection of LVEF [24]. The role of 2D LV GLS has increased and it is currently used as a parameter for defining cardiotoxicity. 3D speckle tracking echocardiography has received much attention recently with the improvement of 3D technology. Recently, 3D strain-based parameters as 3D LVGLS have been increasingly used in research [25].

Santoro et al., ran a study on 100 consecutive breast cancer patients without cardiac symptoms who received anthracycline and cyclophosphamide and/or 5-fluorouracil for 3–4 cycles. Patients underwent at least two echocardiography evaluations, the first at the time of diagnosis and the second after completion of the anthracycline course.

2D and 3D STE were performed and analyzed according to standardized procedures [26]. Offline quantification of 2D derived GLS, 3D volumetric, and 3D STE analysis including calculation of 3D VGLS, left ventricular global circumferential strain (GCS), left ventricular global area strain (GAS), and left ventricular global radial strain (GRS). More than 15% reduction of GLS between post-treatment exam and baseline was indicative of subclinical cardiotoxicity according to the ASE/EACVI Expert Consensus [27].

They reported that the deterioration of LV myocardial mechanics included all the strain components, with the greatest effect for GCS and GAS. They also reported that the suboptimal feasibility of 3D echocardiography—and in of 3D STE—emerges as a main disadvantage limiting the routine applicability of this technique in breast cancer patients [10].

Zhang et al. in a prospective study included 142 women with breast cancer who received doxorubicin (240 mg/m2) with or without trastuzumab underwent 3D STE at standardized intervals prior to, during, and annually after chemotherapy, left ventricular ejection fraction (LVEF), global circumferential strain (GCS), global longitudinal strain (GLS), principal strain, twist, and torsion were quantified. After a median 2.1 years follow-up period they reported that 3D LVEF, GCS, and GLS, had initial decrement that was followed by modest improvement, though at 2 years these values remained worse than baseline. In contrast, values for 3D twist and torsion plateaued at six months without substantial subsequent change [11].

Chen et al. conducted a study on 89 breast cancer patients who received a 6‐cycle epirubicin plus cyclophosphamide chemotherapy regimen. They divided patients into four groups according to cumulative epirubicin doses: (group 1 with 0 mg/m2 before chemotherapy), (group 2 at early chemotherapy with a dose of 120 mg/m^2^), (group 3 at mid-chemotherapy with a dose of 240 mg/m^2^) and (group 4 at the end of chemotherapy with a dose of 360 mg/m2). GAS, GLS, GCS, and E/A were significantly reduced during mid chemotherapy and at the end of chemotherapy. They reported a significant negative correlation between GAS and anthracycline doses. A GAS of − 31.5% was used as the cut-off value for diagnosing left ventricular systolic dysfunction after receiving chemotherapy. The sensitivity of the previous parameters was 81.9%, and the specificity was 80.3% [28].

Cruz et al. ran a sub-analysis of a single-center prospective observational study of patients with breast cancer undergoing chemotherapy with anthracyclines between August 2011 and August 2018. For this analysis, patients with 3D STE data available before initiation, during and after chemotherapy treatment were selected. One hundred and five breast cancer patients were included, and echocardiographic assessment was done during anthracycline therapy. STE was used to calculate 2D GLS, 3D GLS, 3D GCS, 3D GRS, and 3D GAS. CTRCD was defined as an absolute decrease in 2D/3D LVEF > 10% to a value < 54% or a relative decrease in 2D GLS > 15%. Twenty-four patients developed CTRCD. All 3D strain parameters during chemotherapy were significantly worse than baseline values. Impaired contractility in the anterior, inferior, and septal walls was detected by 3D strain regional analysis. A higher incidence of CTRCD was observed with variations in the 3D GRS and 3D GCS, and 3D GRS variations were an independent predictor of CTRCD. Variations in 3D GCS and 3D GRS had good discrimination for predicting CTRCD, with optimal cut-off values of − 34.2% for 3D GCS and − 34.4% for 3D GRS [29].

Xu et al. enrolled 95 women with breast cancer who received epirubicin (360 mg/m2) and underwent 3D STE at baseline, at the end of chemotherapy, and at 12 months after chemotherapy. RV ejection fraction, LV global longitudinal strain (GLS), RV GLS, and RV free wall longitudinal strain were all assessed using 3D STE. An absolute decrease in 3D LVEF of more than 10% to a value less than 50% was defined as cardiotoxicity, while subclinical CTRCD was defined as a reduction in 3D LV GLS of more than 15%. They reported that subclinical CTRCD occurred in 10.5% of patients during follow-up. The 3D GLS of the LV and the GLS and free wall longitudinal strain (FWLS) of the RV both significantly decreased 12 months after treatment compared to baseline. 3D RV GLS and RV FWLS variations had good discrimination for predicting subclinical cardiotoxicity. Only one independent predictor of subclinical CTRCD was the variance of 3D RV FWLS [30].

Wang et al. made the RV their scope and included 64 BC patients who were hospitalized to receive pirarubicin chemotherapy. All patients were evaluated by 3D strain assessment of the RV, and RV GLS, RV GCS, RV GRS and RVGAS were calculated before and after chemotherapy. They reported that RV GLS and RV GAS were significantly reduced after chemotherapy although the conventional parameters for assessment of RV function like TAPSE were not significantly affected [31].

Guan et al. enrolled 79 breast cancer patients receiving different tumor treatment regimens divided into three groups: G1 received anthracycline-free and nontargeted regimens, G2 included patients treated with anthracycline-containing regimens without targeted therapy and G3 included patients treated with anthracyclines combined with targeted drugs. CTRCD was defined as an absolute reduction in left ventricular ejection fraction (LVEF) of > 5% to < 53%. All patients underwent echocardiography before chemotherapy (T0) and after the end of chemotherapy cycle 2 (T2), cycle 4 (T4), cycle 6 (T6), and cycle 8 (T8). They reported that in comparison with the basic condition before chemotherapy, 3D-GLS, 3D-GCS, 3D-GAS, and 3D-GRS were significantly decreased. The decrease in 3D-GLS and 3D-GCS in G2 and G3 was more obvious than that in G1, the changes were statistically significant in T6, and 3D-GAS and 3D-GRS were significantly changed in T8. The changes in 3D-GLS, 3D-GAS, and 3D-GRS in the G3 group were significantly higher than those in the G1 and G2 groups (*p* < 0.05). The degree of decrease in 3D-GCS was G3 > G2 > G1 [32].

Piveta RB., et al. prospectively studied 51 female patients with breast cancer receiving four chemotherapy cycles with a 21-day interval between them. In each cycle, 60 mg/m2 of non-liposomal doxorubicin was administered, totaling 240 mg/m2, together with 600 mg/m2 of cyclophosphamide, totaling 2,400 mg/m2. Human epidermal growth factor receptor 2 (HER-2) positive patients received trastuzumab treatment.

Patients underwent a comprehensive echocardiographic study during the following 5 stages: baseline, after cumulative doses of 120 and 240 mg/m2 of doxorubicin, and then, after 6 months and at least 1 year after anthracyclines. Cardiotoxicity was defined as a decrease in LVEF by more than 10 percentage points to a value lower than 53%. They reported that the 2DGLS presented changes only at the end of the protocol, after the 240 mg/m2 dosage with no change after a lower cumulative dosage of doxorubicin. By 3DSTE they reported changes in most myocardial deformation parameters: 3D GLS, 3D GRS, 3D GCS, and 3D GAS after the cumulative dose of 240 mg/m2 of doxorubicin and no changes in rotation, torsion, or twist in any of the evaluation stages, alternatively, following a lower cumulative dose of doxorubicin (120 mg/m2), the only variable that significantly shifted was 3D GAS, so they decided that The 3D GAS early changed to below normal values was the only parameter that was associated with the subsequent decrease in LVEF during follow-up, while the other variable indices of LV mechanics derived from 2 and 3D speckle tracking analysis, as well as the conventional echocardiographic data were not predictors of CTRCD, another point they highlighted in their study was the proof of accurate reproducibility of 3DSTE, especially in 3D GCS and 3D GAS, demonstrating their dependability and accuracy indices in the assessment of ventricular mechanics [33].

### 3D speckle tracking echocardiography in non-breast cancer patients

Not only breast cancer and its chemotherapy were the focus of this systematic review, but we were also looking at complementary data for other patients with heterogeneous malignancies who received different chemotherapies.

### Pediatric cancer survivors

Hong-kui Yu et al. ran a study on 53 anthracycline-treated pediatric cancer survivors (mean age, 18.6 years) who had finished treatment for more than a year in comparison to 38 control patients. Three-dimensional speckle-tracking echocardiography was performed to assess 3DLV global and segmental strain, time to peak segmental 3D strain, LV torsion, LV systolic desynchrony index (SDI), global performance index (GPI), and ejection fraction.

Cancer survivors had significantly reduced LV strain parameters globally (torsion, GPI, and greater SDI) and regionally (less affecting basal segments) when compared to the control group. Global 3D strain, SDI, and GPI were correlated with cumulative anthracycline dose. GPI had sensitivity of 84.9% and specificity of 81.6% with cut-off 10.6°/cm to differentiate patients from controls [34].

Li et al. ran a case control study included 83 cancer survivors (43 males) after anthracycline therapy who had been off treatment for more than 5 years and 42 age- and sex-matched healthy subjects (21 males). Acute lymphoblastic leukemia was the most frequent cancer diagnosed in 37 out of the 83 survivors.

Survivors of both sexes had significantly lower 3D GLS, GCS, GRS, GAS, and LV ejection fraction compared with controls. The absence of sexual dimorphism in these survivors was considered a novel finding. Among 3D strain parameters, GLS is considered the most sensitive parameter in the detection of subclinical cardiotoxicity in survivors with the greatest area under the ROC curve. A 3D STE-derived GLS correlated positively with LV ejection fraction and showed 85.7% sensitivity and 80.7% specificity at a cut-off 16.4% in differentiating survivors from controls [35].

### Non breast cancer tumors with different chemotherapy regimens

Miyoshi T et al. retrospectively compared 50 anthracycline treated patients with 20 matched healthy volunteers as a control group. Of the 50 patients, 22 had non-Hodgkin lymphoma, 7 had leukemia, 6 had Hodgkin lymphoma, 4 had breast cancer, 2 had osteosarcoma, and 9 had others.

2D and 3D strain analysis including 3D LV GLS, GCS, GRS and GAS was performed for both groups and they found that while 3D GRS, 3D GLS, and other 2D speckle tracking parameters were not affected significantly in anthracycline group, only 3D GAS and peak 3D global circumferential strains of the anthracycline group were significantly worse than those of the control group and 3D GAS was the only parameter that could be independently linked to the cumulative doxorubicin dose [36].

Mornoş C et al. ran an observational prospective study on 59 anthracycline treated patients, of these patients 26 had breast cancer, 12 had non-Hodgkin’s lymphoma, 10 had Hodgkin’s lymphoma, 8 had acute lymphoblastic leukemia, 2 had acute myeloblastic leukemia, and 1 had osteosarcoma. 3D echocardiography strain parameters and cardiac biomarkers were assessed before and after 12 and 36 weeks of treatment. A reduction in LVEF by ≥ 5% to < 55% with symptoms of heart failure, or an asymptomatic reduction of LVEF by ≥ 10% to < 55% was indicative of subclinical cardiotoxicity. Eight individuals (13.5%) had cardiotoxicity at the end of follow up according to the criteria. While at 12 weeks after chemotherapy 3D GLS, 3D GCS, and 3D GRS revealed significant changes and 3D GLS emerged as the only independent predictor of later cardiotoxicity [37].

Song, F, et al. prospectively studied 101 patients with diffuse large B-cell lymphoma between December 2012 and August 2015, who had been treated with anthracycline. All patients received 4 cycles of R-CHOP treatment. 3D-STE was done at baseline, after the completion of two cycles and four cycles of the regimen. The study showed that LV apical rotation, basal rotation, twist, and torsion significantly reduced compared with strain values at baseline, two cycles and after four cycles of the regimens. Furthermore, apical-basal rotation delay increased significantly after two cycles as well as after four cycles of the regimen [38].

Another prospective observational study by Song F et al. included 89 diffuse non-Hodgkin lymphoma patients receiving treatment that contained anthracyclines every 21 days for a maximum of eight cycles. 2D and 3D STE were performed and analyzed according to standardized procedures. 3D STE analysis included 3D LV GLS and GCS and RV GLS. All parameters were analyzed at baseline, after four cycles and at the end of the regimen. 3D LV GLS, GCS and RV GLS decreased significantly after four chemotherapeutic cycles, while 2D GLS and GCS of LV showed significant deterioration only at the end of treatment and 2D GLS of RV showed no significant variation during the whole study. Hs-cTnT correlated significantly with 3D GLS of LV and with 3D GLS of RV. The cut off value for 3D LV GLS was − 20.4% with a sensitivity of 81% and specificity of 66% and was − 21.9% for RV GLS by 3D with a sensitivity of 71% and specificity of 74% for differentiating patients after therapy from baselines [39].

Guang-Li Hou et al. conducted a study on 58 lung cancer patients who received pemetrexed and cisplatin chemotherapy and compared them with 50 cases as a control group. 3D GAS after three cycles of the chemotherapy group was significantly lower than that of the control group and changes in 3D GAS correlated with oxidative stress and apoptosis markers [40].

Xu et al. in a retrospective observational single center study evaluated sixty patients with diffuse large B-cell lymphoma who received R-CHOP chemotherapy every 21 days for a maximum of eight cycles. Three-dimensional left ventricular strain parameters were measured at baseline, after two and four cycles of the regimen respectively.

There was a statistically significant difference between 3D-GLS at baseline and at the end of four cycles of anthracycline chemotherapy, while there was no significant reduction after completion of the first two cycles, and no significant changes in 3D GCS during the whole study. All longitudinal strains in the middle and apical segments showed a significant decrease after four cycles with an early drop of apical anterior and septal wall LS after two cycles. Serum hs-cTnT levels, as the gold standard diagnostic tool for myocardial injury were significantly increased after two and four cycles, and serum hs-cTnT levels were correlated with 3D-GLS as well as the mean value of 3D-LS of different myocardial segments [41].

Alam et al. in a prospective, single center, observational study included 46 patients diagnosed with different malignancies who received a cumulative dose of doxorubicin of less than 550 mg/m2 from December 2017 to November 2018. Echocardiographic examination was done at baseline and after completion of four cycles of anthracycline based chemotherapy. Most of the patients had breast cancer (40 patients).

In 2D echocardiography, there was no statistically significant difference pre and post chemotherapy regarding EF, however of all 2D strain parameters only 2D GLS showed statistically significant difference with mean percent change of 18.33%.

All 3D strain parameters were observed to be significantly reduced during follow up. The reduction of 3D global longitudinal strain by 29.19%, circumferential strain by 30.65%, area strain by 21.61%, and radial strain by 29.66% after completion of chemotherapy. EF was significantly reduced by 3D echocardiography, but no patient had a fall of EF to less than 50% by either 2D or 3D [42].

Zhao et al. in a prospective observational single center study enrolled 74 patients with diffuse large B-cell lymphoma who received 6 cycles of intravenously anthracycline-based treatment between May 2014 and May 2015. All patients underwent echocardiography at baseline, after the completion of two, four and six cycles of the chemotherapy regimen respectively. A relative reduction of > 10% in 3D RVEF or a relative reduction of > 5% to a value of < 45% was the main definition of RV cardiotoxicity.

3D strain parameters in the form of longitudinal free wall strain RV LFS and longitudinal septal strain LV GLS were statistically significant before and after 4 cycles of chemotherapy and only RV LFS was associated with subsequent RV cardiotoxicity with RVEF decline at the end of follow-up. However, RV LSS and LV GCS were only significantly decreased at T3, suggestive of a late change. Of note, the results showed no significant difference in LVESV, and LVEF from the values at T0 to T3 [43].

Ugur Nadir Karakulak et al. conducted a cross-sectional and observational study on 37 patients with chronic myeloid leukemia, 17 were under dasatinib and 20 were under nilotinib after imatinib failure compared with 50 healthy individuals as a control group. 4D speckle tracking echocardiography derived global longitudinal (GLS), circumferential (GCS), radial (GRS), and area (GAS) strain indices were calculated for patient and control groups.

Myocardial deformation parameters did not show any correlation with demographic, clinical, and laboratory characteristics while there is significant reduction in area, longitudinal, and circumferential strain values with no difference between dasatinib and nilotinib groups. Additionally, area and radial strain had a stronger association with the duration of dasatinib treatment [44].

Diana Mihalcea et al. included 110 patients with non‐Hodgkin’s lymphoma scheduled for CHOP chemotherapy from January 2014 to October 2018 in a prospective observational study. 3D myocardial deformation parameters were evaluated at baseline, after third cycle and chemotherapy completion. Only 18 patients (16%) developed cardiotoxicity as defined by guidelines (3D LVEF decrease < 50%, with > 10% from baseline) after the 3rd cycle, while 3D strain echocardiography showed a significant decrease of all deformation parameters LS, CS, RS, and AS in the study group after 3rd cycle and persistent after final cycle of therapy, with more important reduction in patient who developed cardiotoxicity. LS were identified as the best independent predictors for 3D LVEF decrease at the end of chemotherapy [45].

Wang et al. in a prospective observational study included 30 colorectal cancer patients with 19 male participants with no history of cardiac problems who received mFOLFOX6 (oxaliplatin + 5- fluorouracil + calcium folinate) for a total of 12 cycles. They measured the 3D STE parameters at baseline, and after cycle 1, 6 and 12 respectively. The 3D STE parameters GLS, GAS, MCI and LVtw decreased after the first cycle of chemotherapy. Increasing cumulative dose of mFOLFOX6 correlated with decreases in left ventricular MCI, GLS, GAS, GCS, GRS, and LVtw, with statistically significant differences between pre- and post-chemotherapy. In particular, the decrease in MCI was found to be the most significant with the highest cTnT correlation. Area under the curve of MCI was larger than other parameters, which may be a more sensitive indicator of 3D-STE parameters [31].

Chen et al. included 39 patients with multiple myeloma who received more than 6 PAD chemotherapy cycles (bortezomib + doxorubicin liposome + dexamethasone, a course of 12 days) from July 2019 to December 2020, with no radiotherapy or chemotherapy in a prospective observational study. History of cardiac disease or severe chronic disease was considered as an exclusion criterion. Echocardiography assessment after the 2nd, 4th and 6th cardiac cycles showed no significant change by 2D echocardiography till the end of all six cycles, while 3D-STI parameters changed significantly in the earlier stages. After 2 cycles of chemotherapy RVGLS was significantly lower than that before chemotherapy. After 4 cycles of chemotherapy, RVGCS, RVGLS, RVGRS, and LVGLS were significantly lower than that before chemotherapy. Furthermore, there was a significant negative correlation among the RVGCS, RVGLS, RVGRS, and LV GLS. Among them, RVGLS has the strongest correlation. The results showed that the dose of doxorubicin was negatively correlated with RVGCS, RVGLS, RVGRS, and LV GLS. They concluded that 3D-STI is more sensitive for early detection of subtle cardiotoxicity and should be routinely used [46].

## Discussion

Recent advancements in caner chemotherapeutics have led to remarkable improvements in cancer survival rates [47]. Cardiotoxicity is a major concern associated with cancer treatment [48], which may manifest as cardiac dysfunction with left ventricular ejection fraction (LVEF) decline of more than 10% or an LVEF value below 50% after chemotherapy. However, LVEF lacks accuracy as a measure for early myocardial injury [6]. By the time LVEF values drop, cardiac damage has already progressed to an irreversible stage [7].

In the 2022 cardio-oncology European guidelines, two-dimensional global longitudinal strain (2D-GLS) was introduced as an index of chemotherapy-induced cardiotoxicity, and numerous studies have verified that a decrease in two-dimensional left ventricular longitudinal strain (2D-GLS) of over 15% after chemotherapy in patients with an LVEF greater than 50% indicates subclinical myocardial injury [9, 12]. Nonetheless, 2D-STE tracks speckles in only two spatial planes. Therefore, only a limited portion of the actual motion can be analyzed using 2D-STE [13].

Three-dimensional speckle tracking echocardiography (3D-STI) strain parameters reflect myocardial deformation in three spatial directions: longitudinal, radial, and circumferential. It overcomes the issue of angle dependence in two-dimensional plane tracking, and hence is anticipated to provide more sensitive parameter for early detection of subclinical myocardial dysfunction [14]. This implies that 3D-STE could overcome the "out-of-plane" limitation of 2D-STE and thus, can be more accurate and reproducible. Therefore, 3D-based LV-STE is emerging as a more attractive tool as it combines the benefits of 3D acquisition, which evaluates all the parts of the heart simultaneously, and less dependence on operators [15].

GLS is a measure of myocardial deformation from base to apex caused by contraction of the longitudinal fibers beneath the endocardium. Longitudinal myocardial movement is essential to left ventricle contraction. GCS is the short axis circumferential shortening of left ventricular myocardial fibers. GRS reflects left ventricular cavity deformation towards the center, indicating thickening and thinning of left ventricular wall during cardiac cycle. GAS is a product of longitudinal and circumferential strain as the left ventricular endocardial surface deforms during systole and diastole. It reflects the change in relative area by combining longitudinal and circumferential shortening effects [16].

All 3D strains, which encompass GLS, GCS, GRS, and GAS, exhibited a significant correlation with subsequent LV dysfunction in individuals undergoing chemotherapy. Notably, of the various 3D strain measurements, 3D-GAS displayed a tendency towards a strong association with detection and follow-up LV dysfunction, this can be related to the fact that GAS is combining both longitudinal and circumferential shortening effects [49].

Segmental myocardial affection was also an interesting finding and could enable earlier detection of cardiotoxicity. Xu et al. found that all longitudinal strains on middle and apical segments decreased significantly after four cycles of chemotherapy, with an early drop in apical anterior and septal wall longitudinal strains after only two cycles. In a separate study, Cruz et al. observed impaired contractility in the anterior, inferior, and septal walls of breast cancer patients using 3D strain regional analysis. These findings suggest that myocardial segments supplied by the left anterior descending artery are more susceptible to anthracycline treatment than other segments, but the underlying mechanism remains unclear. Potential factors contributing to this susceptibility may include differences in shear stress and left ventricular geometry, greater anthracycline exposure in end-stage circulatory areas, fibrosis, or local variations in apoptotic activation. Further research is still needed to fully understand the mechanisms behind segmental cardiotoxicity in patients undergoing anthracycline treatment.

### Role in the follow up in cancer survivors

Incorporating 3D myocardial strain imaging into the follow-up strategy helps in early detection of subclinical cardiotoxicity.

In two studies by Hong-kui Yu et al. and Li et al. included pediatric cancer survivors; the area under ROC curve showed that 3D-derived GLS is more sensitive than other 3D and 2D strain measures in the evaluation of chemotherapy -induced cardiotoxicity. Potential advantage may be explained by the simultaneous evaluation of myocardial deformation in several LV myocardial segments and the tracking of speckle motion in a real 3D manner [34, 35].

### RV cardiotoxicity evaluation

While most studies focused on LV evaluation, few studies focused on RV evaluation in early detection of subclinical cardiotoxicity. There is growing evidence to suggest that the right ventricle is more susceptible to chemotherapy-induced cardiotoxicity than the left ventricle [50]. Chemotherapy related toxic effects on the right ventricle have drawn attention because of the recognized prognostic significance of RV dysfunction on outcome of patients with heart failure, [18, 51]. Unique anatomical and functional characteristics of the RV that make it more vulnerable to damage (RV is a thin-walled, crescent-shaped chamber that is more vulnerable to pressure and volume overload and RV is exposed to greater fluctuations in pulmonary pressures than the LV) which can make it more liable to toxic effects and earlier changes [52].

However, the available evidence on the impact of chemotherapy treatment on right ventricular 3D strain parameter is limited, only 4 studies reported the outcome on GLS and showed nonsignificant pooled effect size of anthracycline treatment on right ventricle and another two studies reported the effect of chemotherapy on global radial strain (GRS) and circumferential strain (GCS). The pooled mean difference for GRS and GCS was lower after chemotherapy treatment but not statistically significant. While these findings provide preliminary insights into the potential impact of anthracycline treatment on right ventricular function, further research is needed to fully understand these effects.

### limitation of the 3D speckle tracking

Although 3D echocardiography images have the advantage of being able to measure LV volume, LVEF, and 3D strain simultaneously, it has low temporal and spatial resolution and requires acquisition of 6 consecutive beats. So, a good acoustic window without arrhythmia is mandatory.

Most of the studies have a good feasibility and reproducibility of 3D STE, only one study has a concern in 3D feasibility in cancer patient receiving chemotherapy. This was highlighted by Santoro et al. who stated that suboptimal feasibility of 3D echocardiography and in particular of 3D STE emerges as a main disadvantage and strongly limits the routine applicability of this technique in breast cancer patients. However, their result may be related to specific group of patients as left side breast cancer, left side mastectomy or radiotherapy that may limit the 3D study.

## Conclusion

3D speckle tracking echocardiography has the utility of non-invasive and objective evaluation of changes in left ventricular function in cancer patients undergoing chemotherapy. This allows for early detection, early intervention before symptoms appear and optimization of chemotherapy protocols.

With continuous advancement in 3D technology, 3D-STI could be used as a monitoring method in assessment of patients receiving chemotherapy and in predicting their prognosis. However, long-term follow-up and more multicenter studies are needed to determine the predictive and diagnostic threshold for post-chemotherapy myocardial toxicity.

## Data Availability

The datasets used and/or analyzed during the current study available from the corresponding author on reasonable request.

## Abbreviations

3D: Three Dimension
2D: Two Dimension
3D-STI: Three Dimensional-Speckle Tracking Imaging
ANT: Anthracycline
ASE: American Society of Echocardiography
BS: Breast Cancer
CTRCD: Cancer Therapy Related Cardiac Dysfunction
E/A: Ratio between E-wave and A-wave
ECAVI: European Association of Cardiovascular Imaging
EF: Ejection Fraction
FWLS: Free Wall Longitudinal Strain
G1: Group 1
G2: Group 2
G3: Group 3
GAS: Global Area Strain
GCS: Global Circumferential Strain
GLS: Global Longitudinal Strain
GPI: Global Performance Index
GRS: Global Radial Strain
HER-2: Human Epidermal Growth Factor Receptor 2
hs-cTnT: High-sensitive cardiac troponin T
LS: Longitudinal Strain
LV: Left Ventricle
LVEDV: Left Ventricular End-Diastolic Volume
LVEF: Left Ventricular Ejection Fraction
LVESV: Left Ventricular End-Systolic Volume
LVGLS: Left Ventricular Global Longitudinal Strain
LVtw: Left Ventricular Twist
MCI: Myocardial Composite Index
mFOLFOX6: Oxaliplatin + 5- Fluorouracil + Calcium folinate
PAD: Bortezomib, Doxorubicin (Adriamycin) and Dexamethasone
PRISMA: Preferred Reporting Items for Systematic Reviews
R-CHOP: Rituximab- Cyclophosphamide, Hydroxydaunomycin, Oncovin, Prednisone
RCT: Randomized Clinical Trials
ROC curve: Receiver Operating Characteristic Curve
RV: Right Ventricle
RVLFS: Right Ventricle longitudinal free wall strain
RVLSS: Right Ventricle longitudinal septal strain
SDI: Systolic Dyssynchrony Index
STE: Speckle Tracking Echocardiography
STI: Speckle Tracking Imaging
TAPSE: Tricuspid Annular Plane Systolic Excursion
